# Unlocking geothermal energy for sustainable greenhouse farming in arid regions: a remote-sensed assessment in Egypt’s New Delta

**DOI:** 10.1038/s41598-023-48667-4

**Published:** 2023-12-12

**Authors:** Anwar Hegazy, Sami Z. Mohamed

**Affiliations:** 1 Department of Mechanical Engineering, Arab Academy for Science, Technology and Maritime Transport, Alexandria, 1029 Egypt; 2 Land and Water Technologies Department, Arid Lands Cultivation Research Institute (ALCRI), City of Scientific Research and Technological Applications (SRTA-City), Alexandria, 21934 Egypt

**Keywords:** Engineering, Renewable energy

## Abstract

This study introduces a novel approach for assessing geothermal potential in arid regions, specifically Egypt’s New Delta Agriculture Mega Project area. The challenge of limited sub-soil temperature profile data was addressed by integrating Global Land Data Assimilation System (GLDAS) weather data. Using the Earth-to-Air Heat Exchanger (EAHE) model, the extracted air and sub-soil temperature profiles the potential for geothermal energy production was estimated. We modeled the annual sinusoidal soil surface periodic heating pattern by utilizing GLDAS ambient air temperature (AAT) and land surface temperature (LST). Using either AAT or LST yielded a Root-Mean-Square Error (RSME) of 0.2°C. The generated sub-soil profiles for the New Delta region showed a temperature variation of no more than 1.5°C at a 4-m depth, making it an optimal depth for EAHE installation. One-pipe EAHE demonstrated a cooling/heating capacity ranging from 400 W (cooling) to −300 W (heating). The study highlights the New Delta region’s strong geothermal potential for greenhouse cooling and heating, underlining its suitability as a sustainable energy source in arid areas. It also offers a practical guide for the EAHE application and it emphasizes the global potential for geothermal energy exploration, using innovative GLDAS data to expand sub-soil temperature profile accessibility.

## Introduction

Climate variability and change significantly impact agriculture, which is a crucial socioeconomic activity worldwide^1^. In Egypt, limited resources pose challenges to achieving food security^2^. However, the country’s strategic location and innovative agricultural techniques offer opportunities for expanding agriculture horizontally^3^. This shift could generate employment, stimulate local economies, and enhance the well-being of communities^4^. The New Delta mega-project, a major initiative in Egypt’s long-term transformation plan, seeks to reclaim 2.3 million acres of land west of the Nile Delta and establish industrial complexes that integrate agricultural production, using state-of-the-art techniques for cultivation, sorting, packaging, and manufacturing^5^. These activities require advanced modular technologies for sustainable infrastructure. Horizontal expansion strategies like this are essential for addressing food security concerns and promoting sustainable agricultural practices that can help mitigate the impact of climate change.

Greenhouse cultivation is a sustainable agricultural practice with significant potential for saving energy and water compared to open-field cultivation, particularly in arid regions^6^. However, to fully realize the benefits of greenhouse agriculture, it is essential to address its challenges. In arid regions, the main challenge is maintaining optimal indoor climate conditions for crop growth, including ventilation rate, temperature, and relative humidity^7^. To address this, given the elevated ambient temperature, research on greenhouses in arid regions has focused on developing sustainable cooling strategies suitable for such climates as highlighted by Ghani et al.^7^. The greenhouse interior condition control technologies are thoroughly discussed in the recently published review article by Soussi et al.^8^. One promising technology that could be implemented is an Earth-Air Heat exchanger (EAHE)^9^. This innovative technology takes advantage of the fact that shallow soil layers up to 5 m remain stable year-round^10^ and could be used to regulate air temperature. The EAHE presents a simple and new approach to sustainable cooling and heating in greenhouses in arid regions, which can maximize their benefits while minimizing their environmental impact.

**Figure 1:**
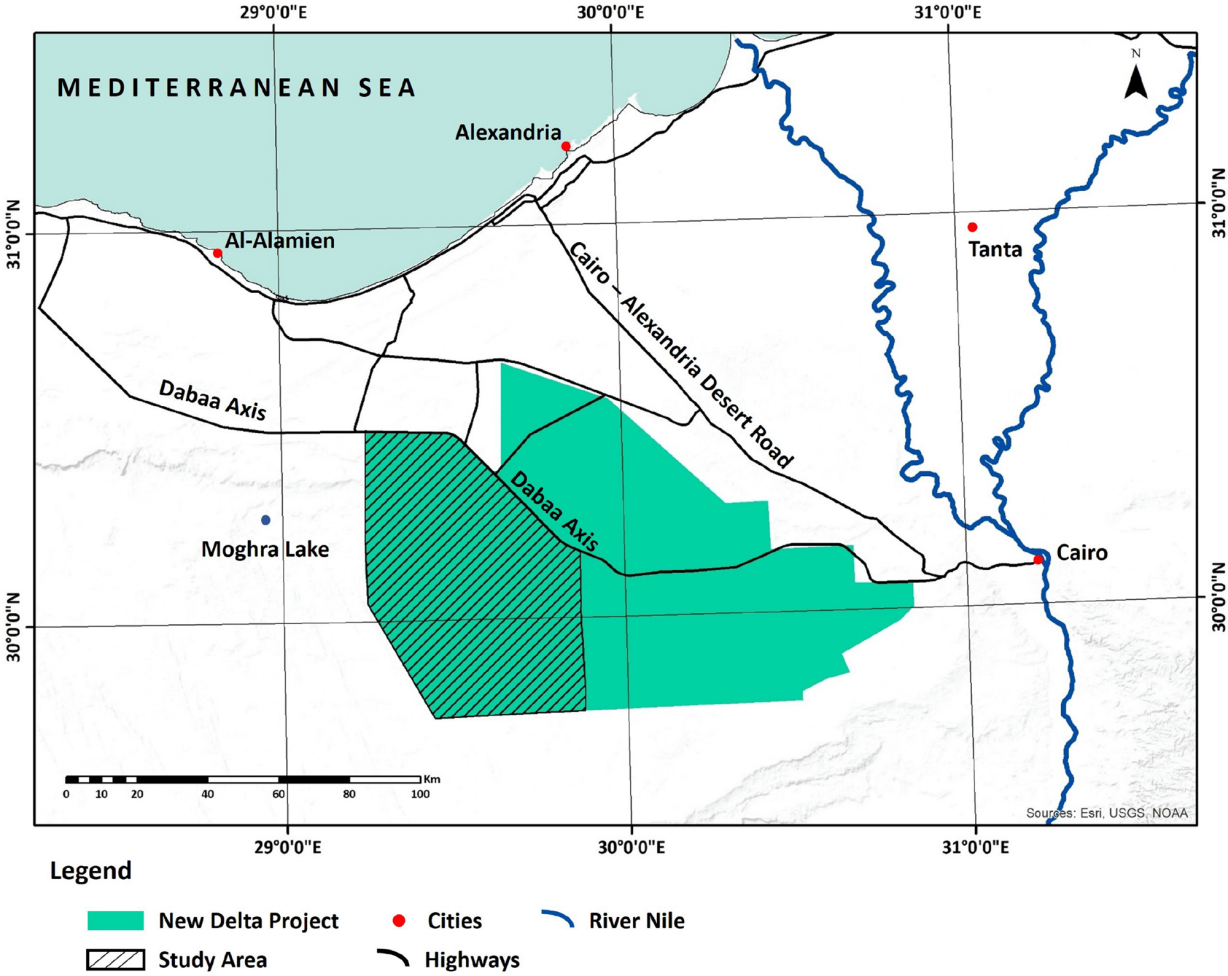
Study area location.

Several studies have investigated the integration of EAHE in greenhouses in arid regions such as Egypt^11^, Australia^12^, Algeria^13, 14^, and Saudi Arabia^15^. These studies have shown that EAHE integration promises significant energy and water savings in greenhouse agriculture. However, one critical step in assessing the performance of EAHE is determining the sub-soil temperature at the installation site. This can be done either through field measurements^16^ or estimated through mathematical modeling^17^. The experimental approach applicability may be limited, suggesting the need for an analytical system presentation to enhance cost-efficiency^18^. Based on the transient heat conduction differential equation and energy balance equation at the ground surface, mathematical modeling has been applied in several studies^19–21^. However, simulating the thermal behavior of the ground as a function of depth and time from a single point is challenging due to various factors such as weather variations, seasonal changes, soil moisture content, and thermal conductivity^22^. To overcome this challenge, Ozgener et al.^22^ proposed a practical approach to predict soil temperature variations by estimating daily soil temperatures based on depth and time, using readily available data such as annual daily average air temperatures. Such an approach is reliable and has been commonly used^23^. Hegazy et al.^12, 24, 25^ used Typical Meteorological Year (TMY) data to estimate sub-soil temperature in arid regions, but this approach requires the availability of weather stations. Similarly, in the research conducted in Iran^26^ the weather data was required to estimate the sub-soil temperature profile. In our study location, as shown in Fig. 1 the nearest cities to the study area that have weather stations are Cairo and Alexandria, where both cities are over 100 km away. The sparse distance makes it challenging to attain weather data for our study location. Furthermore, as stated on the World Meteorological Organization (WMO) website^27^, the weather data for Egypt sourced from the Egyptian Meteorological Authority (EMA) relies on monthly averages spanning the 30-year period from 1981 to 2010, indicating that this information is now a decade old. The limited data availability poses a significant challenge for using shallow geothermal energy, particularly in areas with limited access to climate and environmental information, such as arid regions, as Abdel-Ghany et al.^16^ and Ceglia et al.^28^ have discussed.

Remote sensing offers a cost-effective solution to acquire climate data in countries with limited ground-based monitoring networks. The Global Land Data Assimilation System (GLDAS) is one of the largest global datasets available, providing crucial information on land surface variables^29^. This system is an offline terrestrial modeling system that was jointly developed by the National Aeronautics and Space Administration (NASA) Goddard Space Flight Center (GSFC) and the National Oceanic and Atmospheric Administration (NOAA)/National Centers for Environmental Prediction (NCEP)^29^. GLDAS offers a unique opportunity for the geoscience community to assess global and regional environmental changes with a spatial resolution of up to 0.25 and a temporal resolution of 3 h. The land surface water states and fluxes that GLDAS provides include rainfall rate, snowfall rate, evapotranspiration (ET), soil moisture and temperature in different layers, surface runoff, and subsurface runoff^30^.

### Scope and objectives

The existing literature has extensively highlighted the advantages of employing EAHE in greenhouse agriculture, particularly in terms of energy and water conservation. However, a crucial prerequisite for investigating the application of EAHE is having accurate sub-soil temperature data at the installation depth. Obtaining this information through experimental means is often cost-prohibitive. Previous research has proposed estimating sub-soil temperatures using mathematical modeling based on available weather data as a viable alternative. However, this approach faces limitations in arid regions, where comprehensive weather data is typically scarce. This critical gap in knowledge is what our study aims to address.

To bridge this gap, we introduce an innovative approach that combines remote sensing data, specifically land surface temperature (LST) data, with a sub-soil temperature profile model and an EAHE model. This integrated approach allows us to evaluate the potential effectiveness of EAHE for greenhouse cooling and heating in regions with limited weather data. To the best of our knowledge, our study represents the first attempt to couple these elements, marking a significant advancement in this field. The study outlines the novel methodology and comprehensively describes the data handling and analysis techniques employed throughout the process.

## Methods

### Study area

The New Delta Agriculture mega project is located in the Western Desert, south of Al-Alamein and west of the Nile delta, as shown in Fig. 1. The total project area is 2.3 million acres, making it the largest in the history of Egyptian agricultural projects^31^. The area is characterized by a warm and arid climate. The average temperature is around 23 °C, and there is limited rainfall, with the most precipitation in the form of scattered thunderstorms in the winter months. The area, in general, is dominated by Neogene and Quaternary-aged sedimentary rocks, including sandstone with dune fields^32^.

### System description

**Figure 2:**
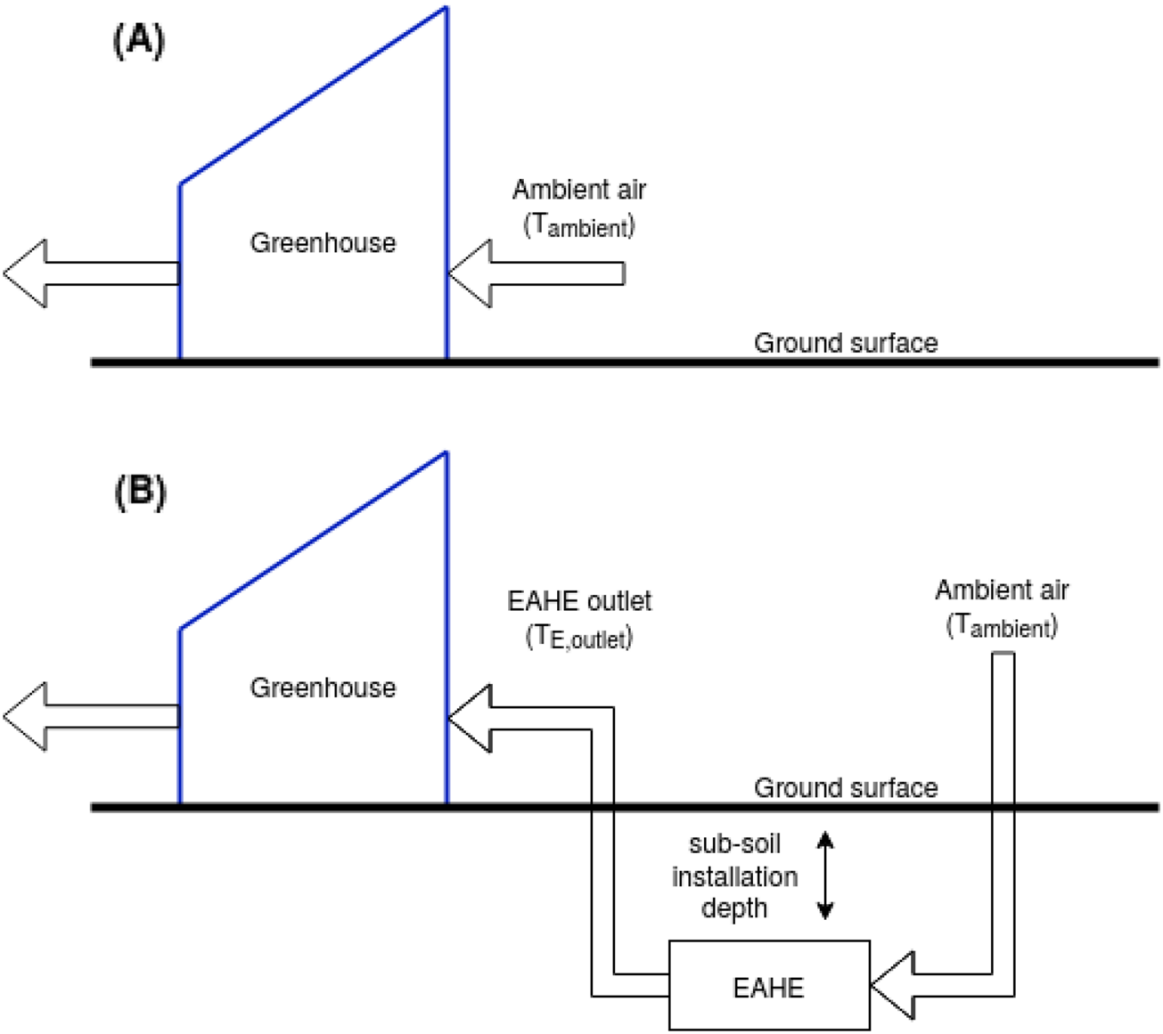
Schematic (**A**) ventilated greenhouse (**B**) EAHE coupled greenhouse.

Greenhouses require cooling, heating, and ventilation to achieve sustainable crop cultivation year-round. In this work, we assess the potential of EAHE in moderating the vent air temperature before directing it to the greenhouse. As shown in Fig. 2, compared to a typically ambient air vented-greenhouse (A) in the EAHE coupled greenhouse (B) the vent air is passed through a single or a set of sub-soil installed PVC pipe/s where it exchanges heat with the surrounding soil. The cooling or heating capacity is dependent on the temperature difference between the inlet and outlet air temperature. Both require climatic data of the location, and the latter requires modelling the heat transfer within the EAHE. Accordingly, we first use satellite remote-sensing (RS) data to collect information on the climate at the study location. Secondly, we describe sub-soil temperature model in “Methods” section, which is input to the EAHE model. After that, we model the heat transfer mechanism between the air and the surrounding soil at the installation depth. As such, using the input air temperature (i.e., ambient) and the EAHE outlet air temperature, we can conclude the output temperature and, hence, the cooling/heating capacity of the EAHE.

### GLDAS data

Remote sensing has become a valuable tool for studying LST, as it provides a synoptic and cost-effective method for mapping and monitoring LST’s spatial and temporal variations. Remote sensing-based LST studies can provide crucial information on the surface energy balance, which is essential for understanding the interactions between the land surface and the atmosphere^33^. The study utilized GLDAS 2.1 to examine trends in LST in the New Delta project area. The data’s high spatial and temporal resolution and the availability of a long period of records made it possible to accurately assess LST trends accurately over time. GLDAS 2.1 data were obtained from the NASA Earth Data portal (https://earthdata.nasa.gov), with a spatial resolution of 0.25° and a 3-h temporal resolution from 2021. GLDAS data were processed to extract the LST data. In order to extract data, the study area was covered by multiple pixels; the average of the 8 pixels covering the study area was calculated for the year 2021. This averaging technique helped to reduce the effects of any individual pixel’s noise or outliers and provided a more reliable and representative estimate of the LST in the region. Using this approach, the study obtained a robust data set that accurately captured the trends in the study area over time.

### Soil particle distribution

In pursuit of a comprehensive assessment of soil particle size distribution as the soil texture highly influences soil temperature regarding pores size, solute transport, holding capacity, conductivity, etc. The sampling was performed in September 2020 and the particle size analysis was done following Ref.^34^ for 89 multi-depth soil samples ranging from 0 to 150 cm, and the results were weighted average for a holistic soil texture view.

### Sub-soil temperature profile model

The sub-soil temperature profile is influenced by the temperature variations along the soil surface. When the temperature pattern is known, the soil temperature variation with depth is considered as one-dimensional heat conduction through a semi-infinite solid domain (i.e ground). The one dimensional form of the heat equation is given as^35^:

1 $$\begin{aligned} \frac{{{\partial ^2}T}}{{\partial {z^2}}}=\frac{1}{\kappa }\frac{{\partial T}}{{\partial t}} \end{aligned}$$

The surface boundary is assumed to follow a cyclic annual temperature variation so the following boundary condition at the surface is considered as a periodic heating pattern in the form of^35^: $T(0,t)=T_{mean}-\Delta T_{surface}\times \cos \omega (t-t_{o})$, with ($T_{mean}$) being the mean LST, ($Amp._{surf}$) is the amplitude of LST variation, and ($t_{o}$) is the phase constant. Theoretically, as depth tends to infinity the interior node is subject to the following boundary condition^35^: $T(\infty ,t)=T_{mean}$. Accordingly the temperature of the soil as a function of time and depth can be calculated from the following equation^25, 36^,

2 $$\begin{aligned} T(z,t)=T_{mean}-Amp._{surf}\times e^{-z\sqrt{\frac{\pi }{365\alpha }}}\times \cos \left[ \frac{2\pi }{365}\left( t-t_{o}-\frac{z}{2}\left( \sqrt{\frac{365}{\pi \alpha }}\right) \right) \right] \end{aligned}$$

where ($\alpha$) and (*z*) are the thermal diffusivity of the soil and the depth respectively; noting that in the equation ($\alpha$) is in the units $m^{2}/day$,

### EAHE model and cooling capacity calculation

The heat transfer mechanism in the pipe is by two regimes: by convection between the air and the pipe’s inner wall; by conduction through the pipe’s inner wall and the surrounding soil. The convection heat transfer is calculated from the Nusselt number^35^,

3 $$\begin{aligned} h_{conv.}=\frac{Nu\times k_{a}}{d_{p,in}} \end{aligned}$$

where ($k_{a}$) is the Air thermal conductivity, ($d_{p,in}$) is the inner pipe diameter, and the Nusselt number (*Nu*). For $Re>2300$ (i.e., turbulent flow) (*Nu*) is calculated from the Dittus–Boelter correlation^35^,

4 $$\begin{aligned} Nu=0.023Re^{0.8}Pr^{n} \end{aligned}$$

where (*n*) is 0.3 and 0.4 for cooling and heating, respectively. For $Re\le 2300$ (i.e., laminar flow)^35^,

5 $$\begin{aligned} Nu=3.66 \end{aligned}$$

The Reynolds number (*Re*) and Prandtl number (*Pr*) inside the pipe are given by,

6 $$\begin{aligned}{} & {} Re=\frac{\rho v_{air}d_{p,in}}{\mu } \end{aligned}$$

7 $$\begin{aligned}{} & {} Pr=\frac{c_{p}\mu }{k_a} \end{aligned}$$

The EAHE is treated as a heat exchanger with constant wall temperature. The NTU-effectiveness ($NTU-\epsilon$) method is applied which gives the following equations^35^:

8 $$\begin{aligned} \epsilon =1-e^{-NTU} \end{aligned}$$

where the effectiveness ($\epsilon$), number of transfer units (*NTU*) and overall heat transfer coefficient (*U*) are given by^35^,

9 $$\begin{aligned}{} & {} \epsilon =\frac{T_{E,inlet}-T_{E,outlet}}{T_{E,inlet}-T(z,t)} \end{aligned}$$

10 $$\begin{aligned}{} & {} NTU=\frac{UA_{p}}{{\dot{m}}_{E}c_{p,a}} \end{aligned}$$

11 $$\begin{aligned}{} & {} \frac{1}{UA_{pipe}}=\left[ \frac{1}{\pi d_{p,in}L_{p}h_{conv.}}+\frac{\ln (\frac{d_{p,out}}{d_{p,in}})}{2\pi L_{p}k_{p}}\right] \end{aligned}$$

where the area of heat transfer is equal to ($A_{p}=\pi d_{p,out}L_{p}$) where ($d_{p,out}$) is the pipe’s outer diameter. Then, for any length of pipe the outlet temperature ($T_{E,outlet}$) is calculated from^35^,

12 $$\begin{aligned} T_{E,outlet}=T_{E,inlet}+(T(z,t)-T_{E,inlet})\times (1-e^{-NTU}) \end{aligned}$$

where *T*(*z*, *t*) is the soil temperature at installation depth. The mass flow rate of air is calculated from,

13 $$\begin{aligned} {\dot{m}}_{E}=\frac{d_{p,in}^{2}}{4}\pi \rho _{a}v_{air} \end{aligned}$$

The following equation calculates the capacity of the EAHE in cooling the vent air,

14 $$\begin{aligned} {\dot{Q}}_{cooling}={\dot{m}}_{E}c_{p,a}(T_{ambient}-T_{E,outlet}) \end{aligned}$$

The cooling capacity is calculated assuming the soil temperature at the installation depth is lower than the ambient temperature. Therefore, a positive value of cooling capacity indicates that air is undergoing a cooling process and a negative sign indicates it is undergoing a heating process. The EAHE model described was validated against experimental measurements as shown in Hegazy et al.^25^ where the model prediction error was only 2.4% indicating the reliability and accuracy of the model. Since we only have the uncertainty in ($T_{E,outlet}$), we calculate the partial derivative of (${\dot{Q}}_{cooling}$) with respect to ($T_{E,outlet}$) and use that to propagate the uncertainty from the Eq. (14), the partial derivative with respect to ($T_{E,outlet}$) is:

15 $$\begin{aligned} \frac{\partial ({\dot{Q}}_{cooling})}{\partial (T_{E,outlet})} = -{\dot{m}}_{E}c_{p,a} \end{aligned}$$

To calculate the uncertainty ($\delta {\dot{Q}}_{cooling}$) in the cooling rate (${\dot{Q}}_{cooling}$), we used the error propagation formula^37^. The formula considers the absolute values of the product of the mass flow rate (${\dot{m}}_{E}$) and the specific heat capacity ($c_{p,a}$) with the uncertainty in the outlet temperature ($\delta T_{E,outlet}$). The equation represents how small changes in ($T_{E,outlet}$) can affect the cooling rate. This method provides the absolute uncertainty in (${\dot{Q}}_{cooling}$). Accordingly, the uncertainty is calculated from,

16 $$\begin{aligned} \delta {\dot{Q}}_{cooling} = \left| -{\dot{m}}_{E}c_{p,a}\right| \cdot \delta T_{E,outlet} \end{aligned}$$

### Assumptions and limitations

The following are assumptions that we consider in our analysis:

- The heat transfer through soil is assumed to be heat conduction through a semi-infinite solid.
- The soil surface temperature follows an annual sinusoidal heating pattern.
- Same soil property up to 5 m depth.
- The pipe wall temperature equals to the sub-soil temperature at the installation depth.
- The EAHE pipe is sufficiently long (50-m^23, 25^) to avoid the effect of near-pipe soil thermal saturation, which could occur from continuous operation and influence the EAHE performance.
- There are no restrictions on land availability.

## Results and discussion

### Soil type classification

Based on the soil structure illustrated in Table 1, the soil classification is Sandy Soil. The soil’s thermal diffusivity ($\alpha$) could be determined from its density, specific heat, and thermal conductivity, where for Sandy Soil, the properties are 1775 kg/m$^{3}$, 840 J/kg K and 0.91 W/mK, respectively^35^.

**Table 1:** Soil particle size distribution (%).

1 mm	0.5 mm	0.25 mm	0.125 mm	0.053 mm	< 0.053 mm
9.91	27.37	41.36	16.49	4.25	0.62

### Ambient temperature, LST, and sub-soil temperature

**Figure 3:**
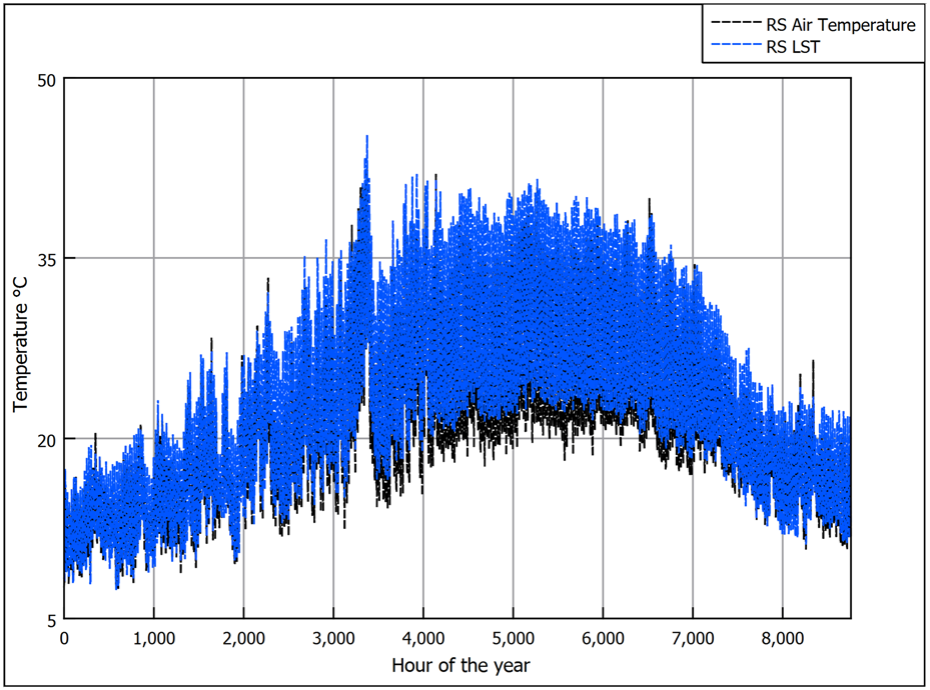
GLDAS data for near surface air temperature and land surface temperature for 2021.

**Figure 4:**
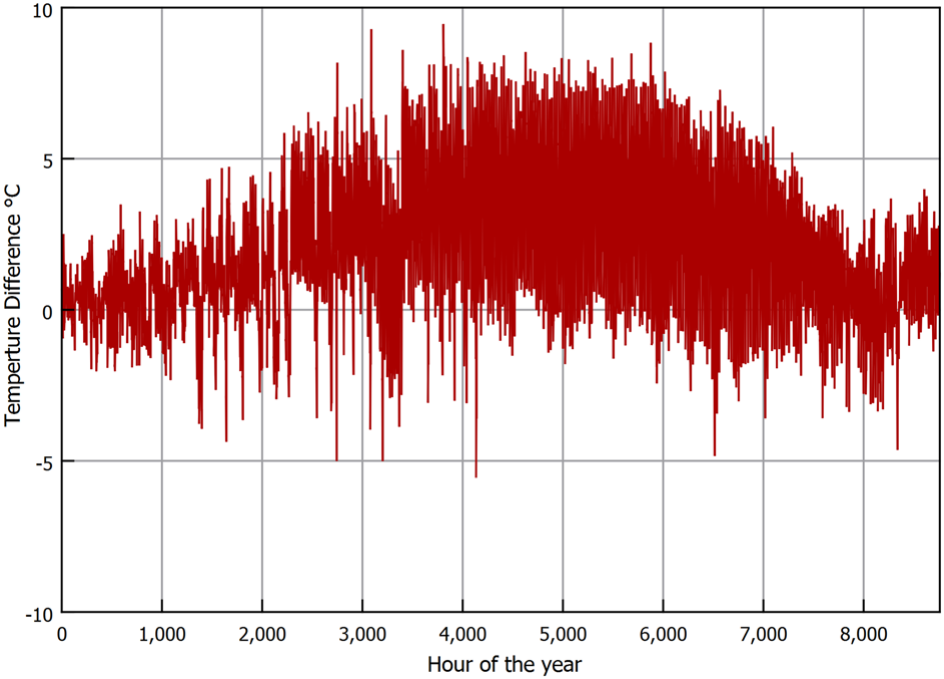
Difference between near surface air temperature and land surface temperature for 2021.

**Figure 5:**
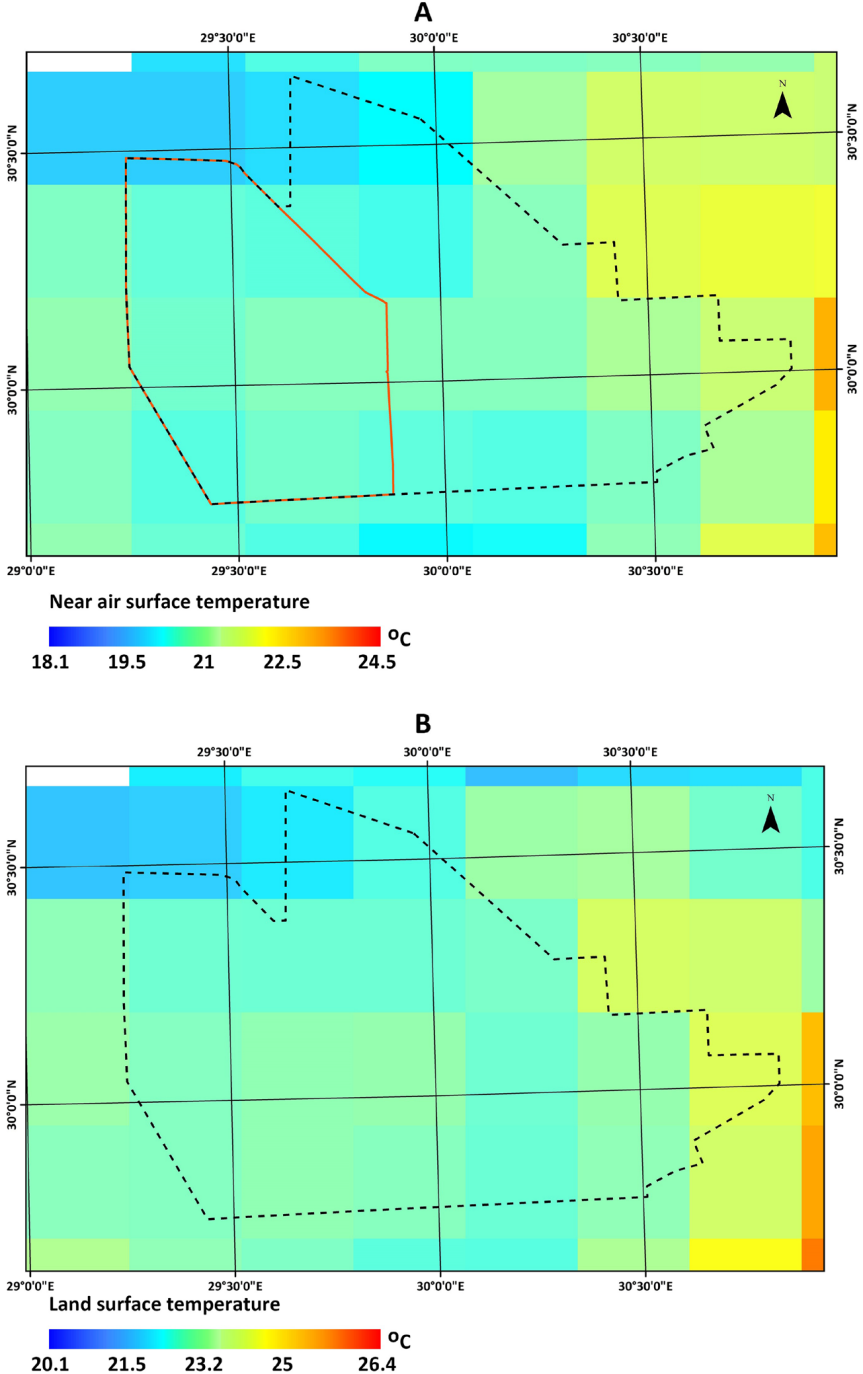
GLDAS 2.1 3-h (**A**) an average of near-surface air temperature and (**B**) land surface temperature for 2021.

**Figure 6:**
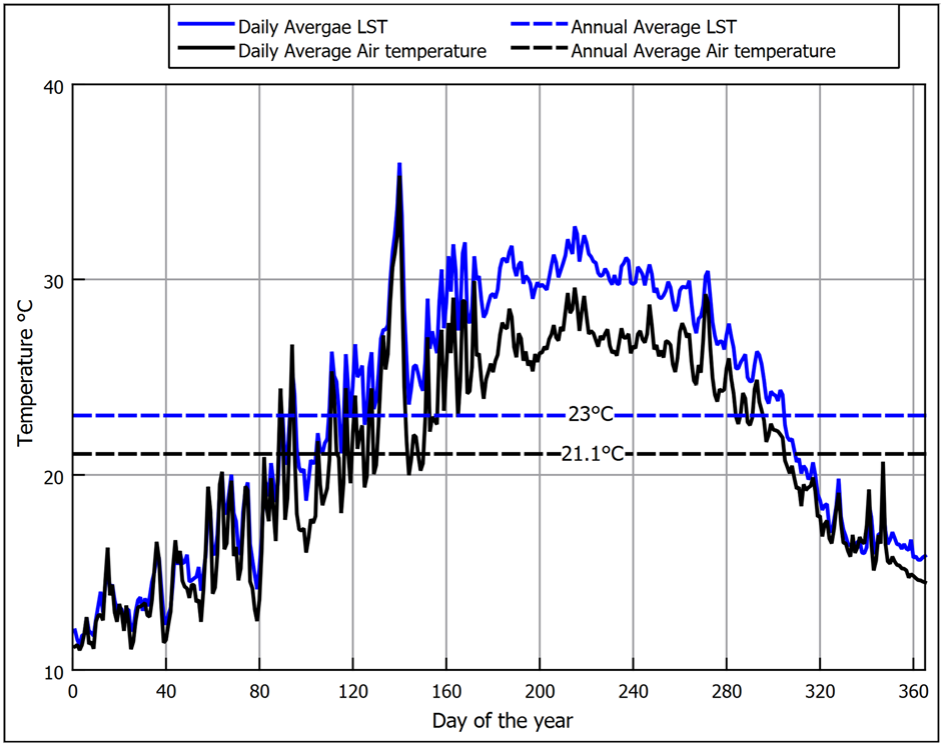
Daily and annual averages for LST and AAT.

Figure 3 shows the ambient air temperature (AAT) (i.e., near surface air temperature typically at 2 m above the ground^38^) and LST collected from NASA satellite RS every 3 h for the year 2021. It can be seen that both temperatures are strongly correlated, where both follow an annual pattern that starts with low temperatures early in the year (i.e., winter) and peaks at mid year (i.e., summer); additionally, it is noted that LST is higher than air temperature, as shown in Fig. 4 that illustrates the difference between the LST and AAT. It was found that the thermal penetration depth of the diurnal LST variation (i.e., morning-night temperature change) is ineffective beyond a depth of 0.5 m^25^ and additionally that the installation depths of EAHE are typically beyond 1 m^23^. Accordingly, the daily average LST over a year are the temperatures to consider to depths of up to 4 m. Hence, to show the temperature trends more clearly, in Fig. 6 we omit the diurnal variation and only show the daily averages for the air and LST together with the annual mean temperatures. We observe that LST is higher than AAT and that the difference is more pronounced in summer. The difference between the annual averages is 1.9°C as seen in Figs. 5 and 6.

As explained previously, the method currently used to estimate the subsoil temperature is conduction through a semi-infinite domain (i.e., soil) that experiences periodic heating.. The periodic heating pattern is influenced by the annual seasonal variation on the surface (i.e., domain boundary). The annual cyclic heating nature follows a sinusoidal pattern as explained by Watson and Labs^36^, where the temperature fluctuates around the annual mean temperature ($T_{mean}$) by a temperature amplitude of ($Amp._{surf}$). The sine wave shifts from the start of the year by ($t_{o}$) days. The daily average LST on each day of the year is required to estimate these variables. One practical method to get this information is by approximating it from the more commonly available annual AAT, given that they are closely correlated^36^. That was the method that Watson and Labs^36^ used to obtain the LST daily variation. From observations in arid regions, the values could be estimated from AAT as follows:

- $T_{mean}$ can be approximated by adding 1.7°C to the average AAT; which is very close to the 1.9°C that we observed in Fig. 6.
- $Amp._{surf}$ can be estimated by adding 1.1°C to one-half the difference between July and January’s monthly average air temperatures.
- $t_{o}$ is calculated according to the periodic heat-conduction theory, where the phase of the solar radiation lags behind the cyclic wave of LST by 1/8 of a cycle or 46 days. Since the day of minimum solar radiation occurs on day 355 of the year, then counting 46 days from that day, the value of ($t_{o}$) is 36, which is the value used in the calculation.

Since we are using RS data where we have both data AAT and LST, we compare using both to estimate the values of $T_{mean}$, $Amp._{surf}$ that are used in Eq. (2). Table 2 shows the values that were extracted from the RS data.

**Table 2:** Values used in Eq. (2).

Variable	Estimation from AAT approximation ($z_0$) (°C)	Estimation from LST ($z_0s$) (°C)
$T_{mean}$	22.7	23
$Amp._{surf}$	8.3	8.75

The values in Table 2 estimated from LST were calculated as follows:

- $T_{mean}$ is the average annual LST.
- $Amp._{surf}$ is one-half the difference between the monthly LST of July and January.

**Figure 7:**
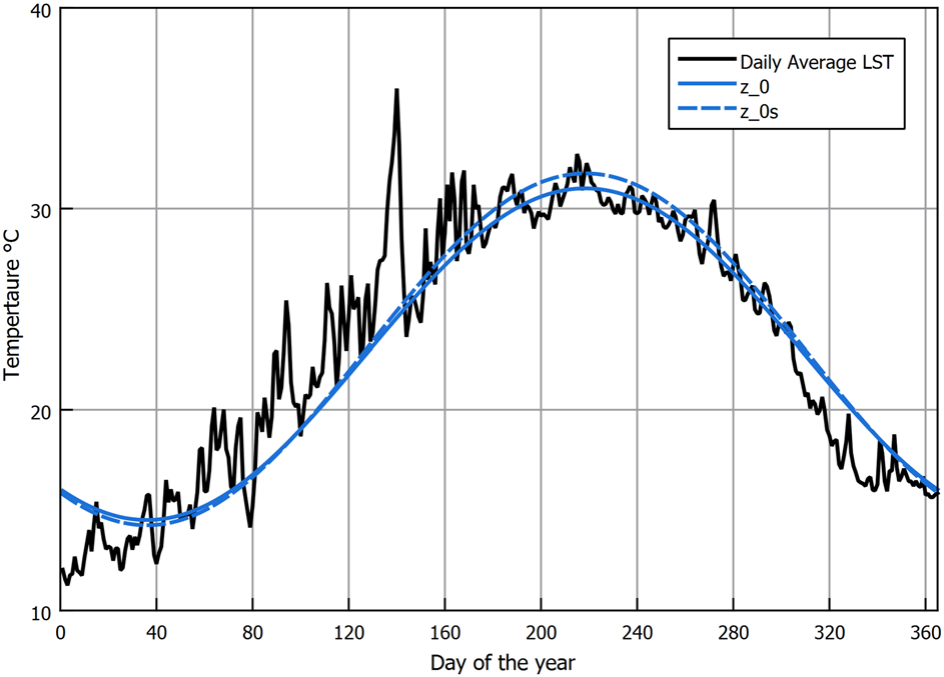
Comparison between daily average LST and mathematically calculated LST using GLDAS RS data correlation.

**Figure 8:**
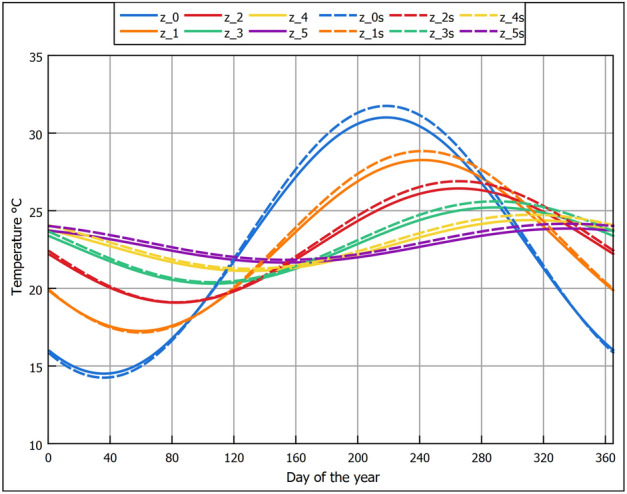
Sub-soil temperature profile at New Delta region.

In Fig. 7, we compare the RS daily average LST values against the mathematical sinusoidal estimation from AAT and LST. It can be seen that the sinusoidal estimation is close to the RS LST values, indicating the reliability of the parametric estimation proposed by Watson and Labs^36^. We note that the estimated values based on LST rather than AAT approximation return higher surface and sub-surface soil temperature values, as seen in Fig. 7. The higher temperatures are caused by the larger ($T_{mean}$) and ($Amp._{surf}$) that are illustrated in Table 2. The approximations from ambient air suggested by Watson and Labs are based on long-term observation in an arid region. Although they are reliable, the correlations might not be exact for different locations. Unless only AAT is available, we suggest that RS LST is a better choice as they are specific to the location. We use the Root-Mean-Square Error (RSME) and the Average Difference (AD) to evaluate the accuracy of a mathematical sinusoidal estimation against the RS LST daily average values. The formulas used were:

17 $${\textrm{RMSE}} = \sqrt{\frac{1}{n}\sum _{i=1}^{n}(y_i - {\hat{y}}_i)^2}$$

18 $$\begin{aligned}{} & {} AD=\frac{1}{n}\sum _{i=1}^{n}(y_i - {\hat{y}}_i) \end{aligned}$$

where (*n*) is the number of data points, ($y_i$) is the RS LST of the i-th data point, and (${\hat{y}}_i$) is the mathematical sinusoidal estimation value of the i-th data point. When using the AAT, approximation the AD was 0.3°C and the RSME was 0.2°C. While for approximation using LST, the AD was 0.05°C, and the RSME was 0.19°C. Despite a minor statistical quality indicator privilege when using LST, the difference between both approaches was insignificant. Therefore, both approaches should be satisfactory depending on the availability of data. Thus, using the values in Table 2 with Eq. (2) the sub-soil temperature at various depths for the New Delta region is shown in Fig. 8. It can be seen in Fig. 8 that at a depth of 4 m, the temperature variation is only 1.5°C which is almost constant year-round. Hence, that depth is a good EAHE installation depth. Comparing sub-soil temperatures in Fig. 8 against the air temperature in Fig. 3, it is seen that the temperature in winter is warmer and vice versa in summer. Therefore, EAHE can be used effectively for temperature moderation all year round.

### EAHE cooling and heating potential

Considering the installation depth of 4 m, we will examine the cooling/heating of EAHE for the two extreme cases, the coldest and warmest hours of the year. Following the parametric recommendations in Hegazy et al.^39^ the design parameters of the EAHE pipe are:

- PVC pipe [density: 1380 (kg/m$^3$)–specific heat: 900 (J/kg K)–thermal conductivity: 0.161 (W/m K)]
- Pipe length 50 m
- air velocity of 2 m/s
- Pipe diameters of 0.1016 m

**Figure 9:**
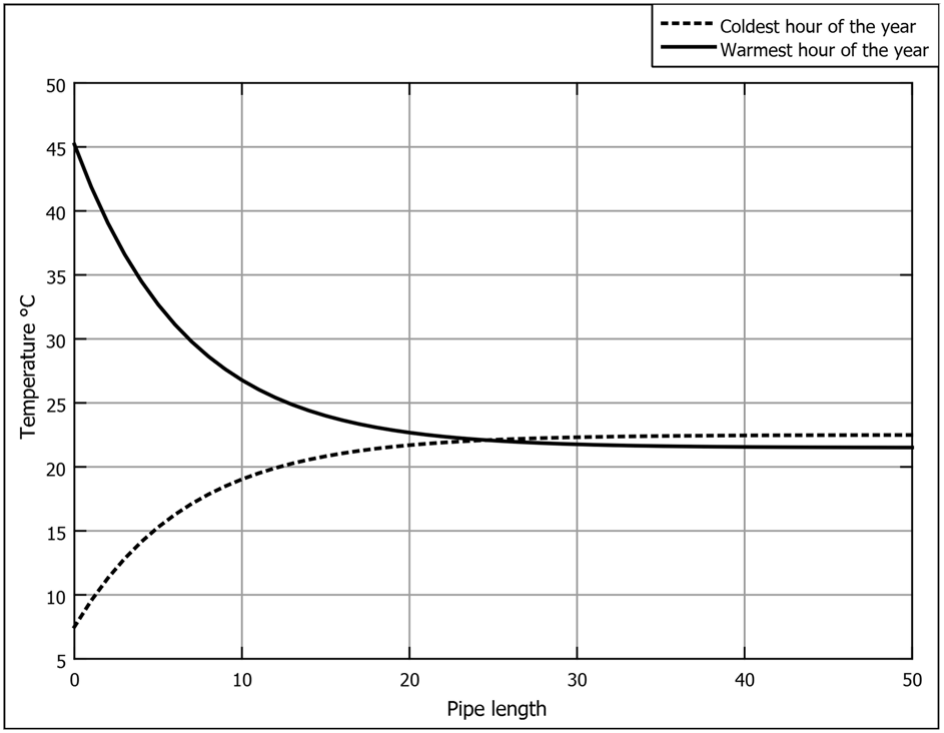
Air temperature variation along pipe length for warmest and coldest hours at 4 m installation depth.

In Fig. 9, we see air temperature evolution with pipe length. For air cooling and air heating processes, there is a steep exponential change in the temperature within the first 10 m of the pipe towards the installation depth sub-soil temperature. The rate of temperature change decreases between 10 and 20 m, then approaches a flat change between 20 and 30 m. The air temperature settles to a constant temperature by the length of 40 m. Nonetheless, the 50-m consideration was a strategy to avoid the effect of near-pipe soil thermal saturation, which could occur during continuous operation^23, 25^. It is evident that using EAHE, the air temperature could be cooled or heated effectively to the stable sub-soil temperature. Therefore, EAHE could provide sustainable cooling, heating, and ventilation to agricultural greenhouses in the New Delta region.

**Figure 10:**
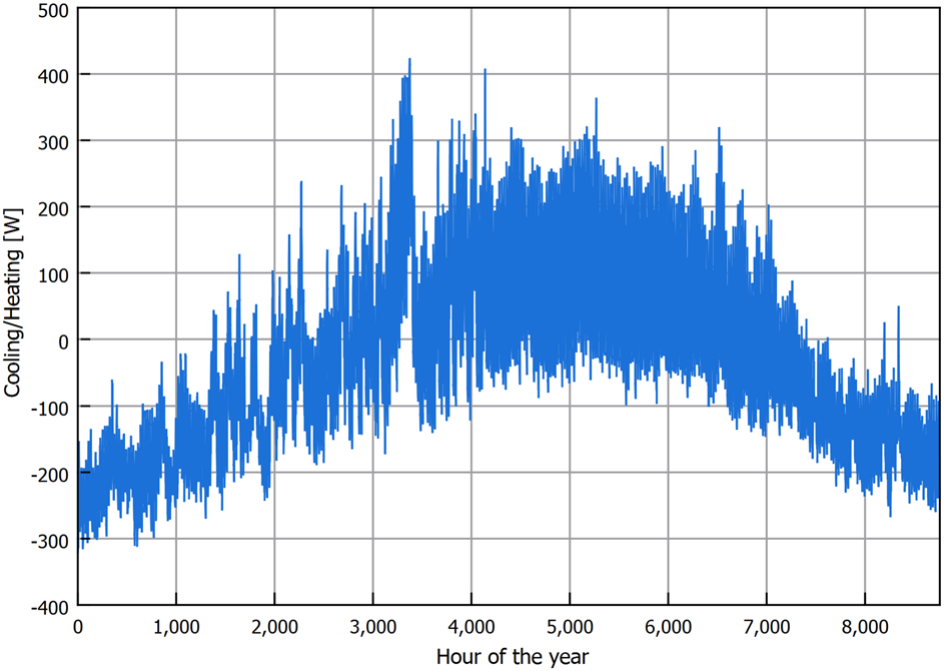
One pipe EAHE cooling potential for an installation depth of 4 m and 50 m pipe length.

The EAHE air outlet temperature is limited to the sub-soil temperature. Following the temperature difference between ambient air and sub-soil temperature that is used for calculating the cooling capacity in Eq. (14), a positive value indicates that air is being cooled, and a negative value indicates air is being heated. Accordingly, the cooling and heating capacities provided by one EAHE pipe are shown in Fig. 10. Following the uncertainty analysis performed in Eq. (16), the uncertainty of the calculated cooling/heating capacity is $\pm \, 0.5$ W resulting from the 2.4% error of the EAHE model prediction. The values shown in Fig. 10 are the hourly energy savings for ventilation air temperature regulation per year that could be provided by one pipe EAHE in the New Delta region. The value varies between the two extremes, 400 W (cooling) and − 300 W (heating), demonstrating the flexibility of EAHE operation in offering cooling or heating to vent air. Additional cooling or heating could be supplied to the vent air after the EAHE outlet by additional systems if required. In such cases, the advantage of EAHE is reducing the energy load on those systems through stabilizing the vent air temperature compared to directly using AAT.

In Fig. 10, we observe that the cooling capacity reaches its minimum point during mid-season, Spring and Autumn. This decline is attributed to the relatively lower temperature difference between the ambient air and the sub-soil compared to Summer and Winter seasons. To optimize energy efficiency during these periods, it is advisable to implement a control system that continually monitors the ambient temperature in relation to the sub-soil temperature. Such a strategy enables precise control of the on-off operation of the EAHE. This approach not only helps conserve energy but also facilitates effective scheduling of maintenance activities for the underground pipes, preventing unnecessary energy consumption associated with air pumping through the EAHE.

## Future work

In order to investigate the potential of geothermal energy, we have integrated remote sensing data with geophysical soil properties and sub-soil temperature thermodynamics using an EAHE. In light of our findings, the following future considerations have been identified:

- That method can be used to investigate any location given the log-lat coordinates and soil sampling test. A generalized shallow geothermal map can be generated, which would be a practical guide to assess the geothermal potential at remote locations with scarce data. This would be helpful for strategic planning in urban, industrial, and agri-land expansion in arid regions.
- A future expansion to this work is considering the diurnal temperature fluctuation effect on the sub-soil temperature up to the depth of 0.5 m, which we did not consider in this investigation.
- Study the effect of climate change on geothermal energy potential.
- Additionally, for shorter EAHE pipes (below the considered 50 m) long-term operation will be considered as it may cause a thermal saturation to the near-pipe soil, which could affect EAHE performance.
- A coupled EAHE and greenhouse simulation will be investigated using RS LST data considering the system dynamics.
- This study will be followed by a detailed economic and environmental evaluation, including excavation and installation costs.
- Similar to recent case studies applied to renewable energy systems in Egypt^40^ and neighboring countries Libya^41, 42^ and Palestine^43^, future investigations should focus on the final Energy saving, the amount of CO2 saving, and the corresponding social cost of the EAHE system.

## Conclusion

This study, which focuses on the New Delta region of Egypt, presents a novel method for investigating the geothermal potential of arid regions. Literature has revealed that the limitation of sub-soil temperature is a challenge at arid regions that constricts the exploration of geothermal energy potential. That issue was solved by extracting weather data from GLDAS for the location of interest. The potential for producing geothermal energy was then calculated using the data of the sub-soil temperature profile and EAHE models.

GLDAS data was employed for both ambient air temperature (AAT) and land surface temperature (LST) to develop an annual sinusoidal heating pattern at the soil surface. Utilizing either AAT or LST resulted in a Root-Mean-Square Error (RMSE) of 0.2°C. Sub-soil temperature profile has been generated and presented for the New Delta arid region, and it was found that the temperature variation at a depth of 4 m was less than 1.5°C, establishing this depth as an ideal choice for the installation of Earth-to-Air Heat Exchangers (EAHE). The results have shown that for the warmest and coldest hours of the year 2021, one pipe EAHE provided cooling/heating capacity that ranged between 400 W (cooling) and − 300 W (heating). The EAHE experiences reduced capacity during mid-seasons, primarily because of the diminished temperature difference between ambient air and sub-soil. Based on this observation, it is advisable to consider using the temperature difference as a key factor in regulating the on-off operation of EAHE and planning maintenance activities.

The findings highlighted the potential for using geothermal energy as a sustainable and renewable energy source in arid regions and showed promising potential for geothermal energy in the New Delta region to provide heating and cooling loads required by greenhouses. The methodology used in this study offers a useful procedure for directly applying EAHE in any setting, opening the door for further research into the global geothermal energy potential. By utilizing GLDAS data, this novel approach could significantly broaden the scope of geothermal energy exploration and increase the accessibility of comprehensive data on sub-soil temperature profiles. Future work has been suggested at the end of the article.

## Data Availability

The data sets used and analyzed during the current study are available from the corresponding author upon reasonable request.
